# Evaluating the quality and equity of patient hospital discharge instructions

**DOI:** 10.1186/s12913-025-12410-8

**Published:** 2025-02-21

**Authors:** Kirsten Austad, Joo Hyun Lee, Howard Lanney, Victoria Oliva Rapoport, Rebecca Wornhoff, Katherine McDaniel, Lindsay Li-Garrison, Brian W. Jack

**Affiliations:** 1 Department of Family Medicine, Boston Medical Center &, Boston University Chobanian and Avedisian School of Medicine, 850 Harrison Avenue, Boston, MA 02118 USA; 2 Evans Center for Implementation and Improvement Sciences, Department of Medicine, Boston University Chobanian and Avedisian School of Medicine, Boston, MA USA; 3 Boston University, Boston, MA USA

**Keywords:** Limited english proficiency, Non-English language preference, Transitions of care, Hospital discharge, Patient education

## Abstract

**Background:**

Written discharge instructions improve patient understanding and self-management after hospitalization. While a small number of studies have evaluated the quality of hospital discharge instructions, none have focused on patients with a non-English language preference (NELP) or looked for potential disparities. Our goal was to compare the quality of patient discharge instructions between those with English language preference and NELP, including whether instructions were in the patient’s preferred language, included all content domains recommended by professional groups, and followed best practices for health literacy.

**Methods:**

We analyzed 200 discharge records from inpatient adult medicine discharges at one hospital across a range of diagnoses using case matching by diagnosis and age to construct an English and NELP cohort (each *n* = 100). We assessed the percentage of discharge instructions written in the patient’s preferred language, measured word count, and calculated readability scores. Lastly, two individual raters used a scale—the Quality of Discharge Instructions-Inpatient (QDI-I) scale—to rate them across six domains of content quality.

**Results:**

Only 8% of patients with NELP received discharge instructions in their preferred language compared to 100% in the English cohort (*p* < 0.001). The mean overall QDI-I score was similar for the NELP and English cohorts (71.1% of perfect versus 71.3% of perfect, *p* = 0.92), but the domain of return precautions was inferior among those with NELP (80.5% of perfect vs. 88.8% of perfect, *p* = 0.013). Instructions in both groups were written at an eighth- to ninth-grade reading level (age 13–15).

**Discussion:**

We found disparities in quality of written discharge instructions for patients with NELP. Recommended next steps include replication of our methods across health systems and larger sample sizes to examine differences between non-English language groups.

**Supplementary Information:**

The online version contains supplementary material available at 10.1186/s12913-025-12410-8.

## Background

The transition from hospital to home is often challenging for patients and their caregivers. Information exchange between inpatient and outpatient providers is notoriously slow, incomplete, and error prone, placing the onus on the patient to understand the events of hospitalization and care plan once home [1–3]. Patient understanding of discharge instructions is necessary for patients to assume self-care responsibilities and is tied to both improved clinical outcomes and patient satisfaction [4, 5]. But studies over the past three decades show that patients often do not have a clear understanding of provider recommendations within even a few days of discharge, and routinely overestimate their understanding, placing them at higher risk for adverse medication events and hospital readmission [6–12].

Written discharge instructions are an evidence-based practice shown to improve patient understanding [4, 13, 14]. Discharge instructions should be tailored to each patient’s needs, document provider recommendations such as medication changes and diet, and be provided in language understandable to the patient [15–18]. Despite their importance, only a handful of studies have evaluated the quality of hospital discharge instructions [5, 9, 19–21]. Most have been limited to the pediatric population [19, 21]—which might underestimate the medical complexity of the adult discharges—and report the presence or absence of certain content domains without judging the quality of the information presented [5, 20].

Providing high-quality discharge instructions for the growing patient population with limited English proficiency (LEP) is challenging [22]. Patients with LEP experience higher hospital readmission rates for diagnoses that require higher levels of self-management (such as congestive heart failure) and are more likely to report post-discharge problems, including confusion about their discharge instructions [23–27]. Yet no studies have systematically examined the quality of written discharge instructions for patients with LEP; lower quality discharge instruction could potentially contribute to inadequate understanding and more adverse outcomes after discharge.

Given this research gap, we sought to evaluate the quality of personalized hospital discharge instructions of a diverse group of patients with LEP. Our primary goal was to compare discharge instructions between patients with an English and non-English language preference (NELP) to identify potential disparities by whether instructions were written in the patient’s preferred language, included all content domains recommended by professional groups, and followed best practices for health literacy. Our secondary goal was to assess differences in quality of discharge instructions by language group and by whether the diagnosis required high intensity or low intensity of self-management (as readmission rates are higher for conditions requiring high self-management). While LEP remains the dominant term in the field, we will use NELP because it reflects language preference, rather than fluency, which is what was measured in this study and used in clinical care.

## Methods

### Study setting

The study was carried out at a single urban academic tertiary care safety-net hospital in New England that serves a large population with NELP. On discharge, patients received an After Visit Summary (AVS), which is generated by the electronic medical record (EMR) Epic. The AVS compiles key discharge information created by the provider including a medication list and changes and a personalized written summary that prompts the provider to explain the cause of admission (primary diagnosis), care instructions, reasons to seek medical attention (“return precautions”), and medication changes. The AVS template is shown in Appendix 1. The AVS also pulls in the date, time, provider type, and address of scheduled post-discharge appointments and information on how patients can contact the hospital with questions after discharge.

### Record selection strategy

The patient’s preferred language is entered in the EMR by registration staff. Preferred language can be updated by clinicians as needed. Using operational data from 2022, we pulled the preferred language field in the EMR to identify the top four non-English languages at the hospital: Spanish, Haitian Creole, Cape Verdean (Portuguese) Creole, and Vietnamese. These four language groups, plus a fifth composed of all other non-English languages, comprised the “NELP cohort”. The “English cohort” included patients who indicated their preferred language was English as recorded in the EMR.

We used case matching to compare discharge records from patients with NELP to matched English-language-preference patients by primary diagnosis. Given previous evidence that readmission rates are higher among patients with NELP with diagnoses requiring high intensity of self-management, we reviewed a list of the most common inpatient diagnoses and classified each as high or low self-management. High self-management diagnoses include those that commonly require complex medication regimens (e.g., COPD exacerbation), monitoring of vital signs (e.g., atrial fibrillation or paroxysmal tachycardia), daily weight measurement (e.g., exacerbation of heart failure), or point-of-care labs (e.g., diabetes), or detailed dietary instructions (e.g., renal failure). We then selected five common “low self-management” diagnoses—sepsis, chest pain, stroke-like symptoms including transient ischemic attack (TIA), low back pain, and pneumonia—to ensure broad applicability to hospitalized patients.

To build our overall study cohort, we first identified all discharges to home of adults (age ≥ 18) from general medicine teams between January 1, 2017 and December 21, 2022. Each discharge was considered independently without eliminating repeat discharges. Discharges against medical advice were excluded. Two discharge records were randomly selected per diagnosis among the five linguistic categories in the NELP cohort. For each discharge in the NELP cohort, a discharge record from the English cohort was matched to it based on primary diagnosis and patient age within 10 years. This process selected a total of 200 discharge records or 20 per diagnosis, including 10 from patients with English language preference and 10 with NELP. Discharge instructions were included in the analysis even if not provided in the patient’s preferred language.

De-identified versions of the AVS and discharge medication list were provided to the study team by the hospital’s Clinical Data Warehouse for Research. In six cases, either the AVS or physician discharge summary was missing, and a replacement case was selected using the above criteria. If discharge instructions were not written in English, they were professionally translated.

### Data analysis

Demographics were abstracted from the EMR and summarized as means for continuous variables (age) and percentages for categorical variables (race, Hispanic origin, and preferred language). Results were compared between English and NELP cohorts using t-test and Fisher’s exact test, respectively.

#### Readability of discharge instructions

Word counts of each of the 200 discharge records were evaluated by an online calculator. Language concordance was a binary outcome indicating if the language of the instructions matched the patient’s preferred language. For reading level, we first edited patient instructions for standardization as explained in Appendix 2. An online calculator was used to determine the reading level per the widely used Flesch Reading Ease score (FRES) [28, 29]. As the data did not follow a normal distribution, we calculated the median and interquartile range (IQR). The same was done using the New Dale-Chall Reading Scale (NDCRS), which was converted into U.S. grade level groupings—45 or above = 4th grade or less (up to age 9), 34–44 = 5th grade to 8th grade (ages 10–13), and 33 or less = 9th grade or above (age 14 and above)—and reported as percentages in each category [30].

We summarized categorical outcomes as percentages and compared between NELP and English cohorts using Fisher’s exact test. Continuous outcomes were summarized as medians and interquartile ranges (IQRs) given lack of normal distribution and compared between language groups using the Mann–Whitney test. For all analyses, a *p*-value of ≤ 0.05 was used as the cutoff for statistical significance.

#### Content of patient discharge instructions

We conducted a literature review to identify instruments that could be used to judge the quality of patient discharge instruction content. All existing options either assessed only for presence or absence of instruction domains or were integrated with process measures [5, 9, 19–21]. As no tools were identified to assess the quality of the content, the lead author drafted the Quality of Discharge Instructions-Inpatient (QDI-I) scale based on existing literature, experience as a hospitalist, and input from the senior author. The QDI-I evaluates content across six domains: primary diagnosis, self-care instructions, return precautions (i.e. reasons to seek medical attention), medication changes, reasons for medication changes, and recommended follow-up. Each domain is scored from 1 (very poor) to 4 (very good) and summed across the six domains for a possible total score of 24; if no medication changes were made, the maximum total score of 20 was then scaled by multiplying by 1.2. For ease of interpretability, the QDI-I score was also expressed as a percentage of a perfect score. For example, a total score of 18 (out of 24) or 3 (out of 4) for a single domain was reported as 75% of a perfect score. The draft QDI-I was pilot tested by three authors and minor changes were made. The QDI-I is included as Appendix 3.

For each of the 200 discharge records, two physician raters independently assigned a QDI-I score for each domain. Disagreements of more than one point were resolved by the lead author (KA), and a mean score was assigned if raters disagreed by one point. For each rater, an overall score was calculated by summing scores across the six domains. Both overall and domain-specific QDI scores were summarized as means and standard deviations and compared between English and NELP groups using the t-test. R (version 2023.06.1 + 524) was used for analysis.

#### Sub-group analyses by language group and intensity of self-management

To assess for differences by language, we also compared results between the English cohort and each of the five language groups individually. We also compared English and NELP cohorts separately within those selected by high- and low-intensity self-management diagnoses. In both cases we applied the same summative statistics and comparative analyses described above.

## Results

### Demographics

Table 1 displays the demographics for the overall cohort as well as a comparison between the English and NELP cohorts. The average age was 71.1 years and was similar between the two groups (*p* = 0.50). The breakdown by sex was nearly equal and did not differ by language (*p* = 1.00). Those with NELP were significantly more likely to identify as Latino (21% versus 4%, *p* < 0.001) and less likely to be White (7% versus 46%, *p* < 0.001 for comparison of all racial categories).

**Table 1:** Demographics of the overall study population and language cohorts

Characteristic	Overall (*n* = 200)	By Preferred Language		
		**English** **(*n* = 100)**	**NELP** **(*n* = 100)**	***p*-value**
Age in years (mean, SD)	71.1 (11.0)	70.6 (11.0)	71.7 (11.0)	0.50
Sex (n, %)				1.00
Female	99 (49.5%)	50 (50.0%)	49 (49.0%)	
Male	101 (50.5%)	50 (50.0%)	51 (51.0%)	
Race (n, %)				< *0.001*
Black/African American	91 (45.5%)	47 (47.0%)	44 (44.0%)	
Asian	21 (10.5%)	0 (0%)	21 (21.0%)	
White	53 (26.5%)	46 (46.0%)	7 (7.0%)	
Other	23 (11.5%)	4 (4.0%)	19 (19.0%)	
Declined	12 (6.0%)	3 (3.0%)	9 (9.0%)	
Hispanic/Latino (n, %)	25 (12.5%)	4 (4.0%)	21 (21.0%)	< *0.001*
Preferred Language (n, %)				< *0.001*
English	100 (50.0%)	100 (100%)	0 (0.0%)	
Spanish	20 (10.0%)	0 (0.0%)	20 (20.0%)	
Haitian Creole	20 (10.0%)	0 (0.0%)	20 (20.0%)	
Cape Verdean (Portuguese) Creole	20 (10.0%)	0 (0.0%)	20 (20.0%)	
Vietnamese	20 (10.0%)	0 (0.0%)	20 (20.0%)	
Other^a^	20 (10.0%)	0 (0.0%)	20 (20.0%)	

Italics indicate those *p*-values that are significant at *p* < 0.05. For categorical variables *p*-values were generated by Fisher’s exact test and for continuous variables the two sample t-test was used

*NELP* Non-English language preference

^a^Other languages include Albanian, Arabic, Chinese/Cantonese, Greek, Hebrew, Italian, Polish, Portuguese, Russian, Somali, and Tigrinya

### Comparison by English and NELP cohorts

Only 8% of patients with NELP received discharge instructions in their preferred language, as compared to 100% in the English language group (*p* < 0.001; Table 2). On average discharge instructions were shorter in the NELP cohort at 178 words compared to 190 words in the English cohort (*p* = 0.032). In both cohorts the raw FRES was nearly identical and corresponded to an eighth or ninth grade reading level. Using the NDCRS grade-level categorization, 62.0% of patients with NELP received instructions at a ninth-grade reading level or higher compared to only 51.0% in the English cohort, but the difference did not reach statistical significance (*p* = 0.15).

**Table 2:** Comparison of content, readability, and quality of patient discharge instructions between NELP and English cohorts

Characteristic	English cohort (*n* = 100)	NELP cohort (*n* = 100)	*p*-value
Language concordant: %, n	100%, 100	8.0%, 8	< *0.001*
Word count: median, IQR	190, 104.25	178, 93.75	0.079
Flesch Reading Ease score (FRES): median, IQR	62.86, 11.12	63.0, 13.68	0.56
New Dale-Chall Reading Scale (NDCRS)			*0.02*
Easier than recommended: 1st—4th grades (%,n)	0%, 0	0%, 0	
Recommended: 5th—8th grades (%,n)	49.0%, 49	38.0%, 38	
Harder than recommended: 9th grade—college (%,n)	51.0%, 51	62.0%, 62	
Overall QDI-I score: mean, SD, %^a^	17.10, 2.72, 71.3%	17.06, 3.25, 71.1%	0.92
I. Primary diagnosis	3.35, 0.64 83.8%	3.37, 0.61 84.3%	0.82
II. Self-care	2.10, 1.08 52.5%	2.07, 1.08 51.8%	0.87
III. Return precautions	3.55, 0.78 88.8%	3.22, 1.06 80.5%	*0.013*
IV. Medication changes	3.14, 1.24 78.5%	3.20, 1.23 80.0%	0.71
V. Reason for medication changes^b^	2.57, 1.08 64.3%	2.78, 1.12 69.5%	0.22
VI. Recommend follow-up	2.42, 0.91 60.5%	2.45, 0.86 61.3%	0.84

For *p*-values comparing English and NELP groups, Fisher’s exact test was used to analyze categorical variables. For continuous variables, Mann–Whitney test was used except for QDI-I outcomes which used the two-sample t-test

*IQR* Interquartile range, *NELP* Non-English language preference, *QDI-I* Quality of Discharge Instruction-Inpatient scale, *SD* Standard deviation

^a^Percentage represents score relative to a perfect score. For example, a raw total score of 18 would be 18/24 = 75%

^b^Reason for medication changes only applied to those changed during admission; if no medications were changed this domain was not scored and the total score was scaled to 24 (by multiplying by 1.2)

The overall QDI-I score in the NELP and English cohorts was similar (17.06 vs. 17.10, or 71.1% vs. 71.3%; *p* = 0.92). On average patients with NELP received lower quality return precautions compared to those with an English language preference (3.22 vs. 3.55, or 80.5% vs 88.8%; *p* = 0.013). Other domains of the QDI-I did not vary significantly between the two cohorts. In both, the highest scoring QDI-I domain was primary diagnosis and the lowest scoring was self-care instructions.

### Comparison by language group

While all NELP groups were less likely to receive language-concordant discharge instructions compared to English speakers, Spanish was the second most common language with 4 cases (20.0%) receiving language-concordant discharge instructions (Table 3). There were no significant differences in the word count among the NELP groups compared to the English cohort (Appendix 4). Analysis of the FRES data showed Vietnamese speakers received easier-to-read discharge instructions than English speakers (67.56 vs 62.86, *p* = 0.026; Appendix 5). By the NDCRS grade-level categorization, most NELP groups have between 60–70% of discharge records in the ninth-grade-and-above group; however, in the Vietnamese group 45.0% of discharge instructions were written at a 9th-grade level or higher compared to 51.0% in the English group (*p* = 0.81).

**Table 3:** Comparison of content, readability, and quality of patient discharge instructions between English cohort and each language group

	***English cohort***	***NELP cohort***				
	***N* = *100***	**Spanish** ***N* = 20**	**Haitian Creole** ***N* = 20**	**Cape Verdean** ***N* = 20**	**Vietnamese** ***N* = 20**	**Other** ***N* = 20**
Language concordant: %, n (*p*-value)	100%, 100	20.0%, 4 (< 0.001)	10.0%, 2 (< 0.001)	5.0%, 1 (< 0.001)	5.0%, 1 (< 0.001)	0%, 0 (< 0.001)
Word count: median, IQR (*p*-value)	190, 104.25	178.5, 79 (0.24)	166.5, 99.25 (0.17)	191, 87 (0.63)	167.5,83.3 (0.20)	160.5, 97.25 (0.47)
Flesch Reading Ease score: median, IQR (*p*-value)	62.86, 11.12	60.66, 8.86 (0.85)	60.74,17.45 (0.38)	59.07,15.27 (0.76)	67.56, 9.62 *(0.04)*	63.79, 7.14 (0.71)
New Dale-Chall Reading Scale (*p*-value)		(0.15)	(0.33)	(0.15)	(0.81)	(0.62)
Easier than recommended: 1st—4th grades (%,n)	0%, 0	0%, 0	0%,0	0%,0	0%,0	0%,0
Recommended: 5th—8th grades (%,n)	49.0%, 49	30.0%, 6	35.0%, 7	30.0%, 6	55.0%,11	40.0%, 8
Harder than recommended: 9th grade—college (%,n)	51.0%, 51	70.0%, 14	65.0, 13	70.0%, 14	45.0%, 9	60.0%, 12
Overall QDI-I score: mean, SD, (*p*-value),%^a^	17.10, 2.72 71.3%	17.47, 2.63 (0.58) 72.8%	15.88 3.77 (0.09) 66.2%	16.30,3.51 (0.34) 67.9%	17.66, 2.86 (0.43) 73.6%	18.02, 3.15 (0.24) 75.1%
I. Primary diagnosis	3.35, 0.64 83.8%	3.53, 0.60 (0.23) 88.3%	3.33, 0.52 (0.88) 83.3%	3.50, 0.61 (0.31) 87.5%	3.25, 0.57 (0.51) 81.3%	3.23, 0.72 (0.49) 80.8%
II. Self-care	2.10, 1.08 52.5%	2.23, 1.24 (0.67) 55.8%	1.83, 0.86 (0.23) 45.8%	2.00, 1.01 (0.71) 50.0%	2.33, 1.15 (0.42) 58.3%	1.98, 1.12 (0.66) 49.5%
III. Return precautions	3.55, 0.78 88.8%	3.40, 0.87 (0.48) 85.0%	2.95, 1.29 *(0.006)* 73.8%	2.88, 1.06 *(0.001)* 72.0%	3.30, 1.12 (0.23) 82.5%	3.58, 0.88 (0.91) 89.5%
IV. Medication changes	3.14, 1.24 78.5%	3.30, 1.14 (0.57) 82.5%	2.93, 1.37 (0.53) 73.3%	2.85, 1.42 (0.41) 71.3%	3.45, 1.02 (0.24) 86.3%	3.48, 1.18 (0.23) 87.0%
V. Reason for medication changes^b^	2.57, 1.08 64.3%	2.67, 1.22 (0.77) 66.8%	2.74, 1.19 (0.60) 68.5%	2.78, 1.03 (0.46) 69.5%	2.68, 1.10 (0.72) 67.0%	3.00, 1.15 (0.16) 75.0%
VI. Recommended follow-up	2.42, 0.91 60.5%	2.30, 0.73 (0.53) 57.5%	2.23, 0.94 (0.40) 55.8%	2.35, 0.76 (0.72) 58.8%	2.60, 0.98 (0.46) 65.0%	2.75, 0.85 (0.13) 68.8%

For categorical variables, *p*-values generated by Fisher’s exact test. For continuous variables, *p*-value generated by Mann–Whitney test except for QDI scores for which the two sample t-test was used

*IQR* Interquartile range, *NELP* Non-English language preference, *QDI-I* Quality of Discharge Instruction-Inpatient scale, *SD* Standard deviation

^a^Percentage represents score relative to a perfect score. For example, a raw total score of 18 would be 18/24 = 75%

^b^Reason for medication changes only applied to those changed during admission; if no medications changed this domain was not score and the total score was scaled to 24 (by multiplying by 1.2)

**Fig. 1:**
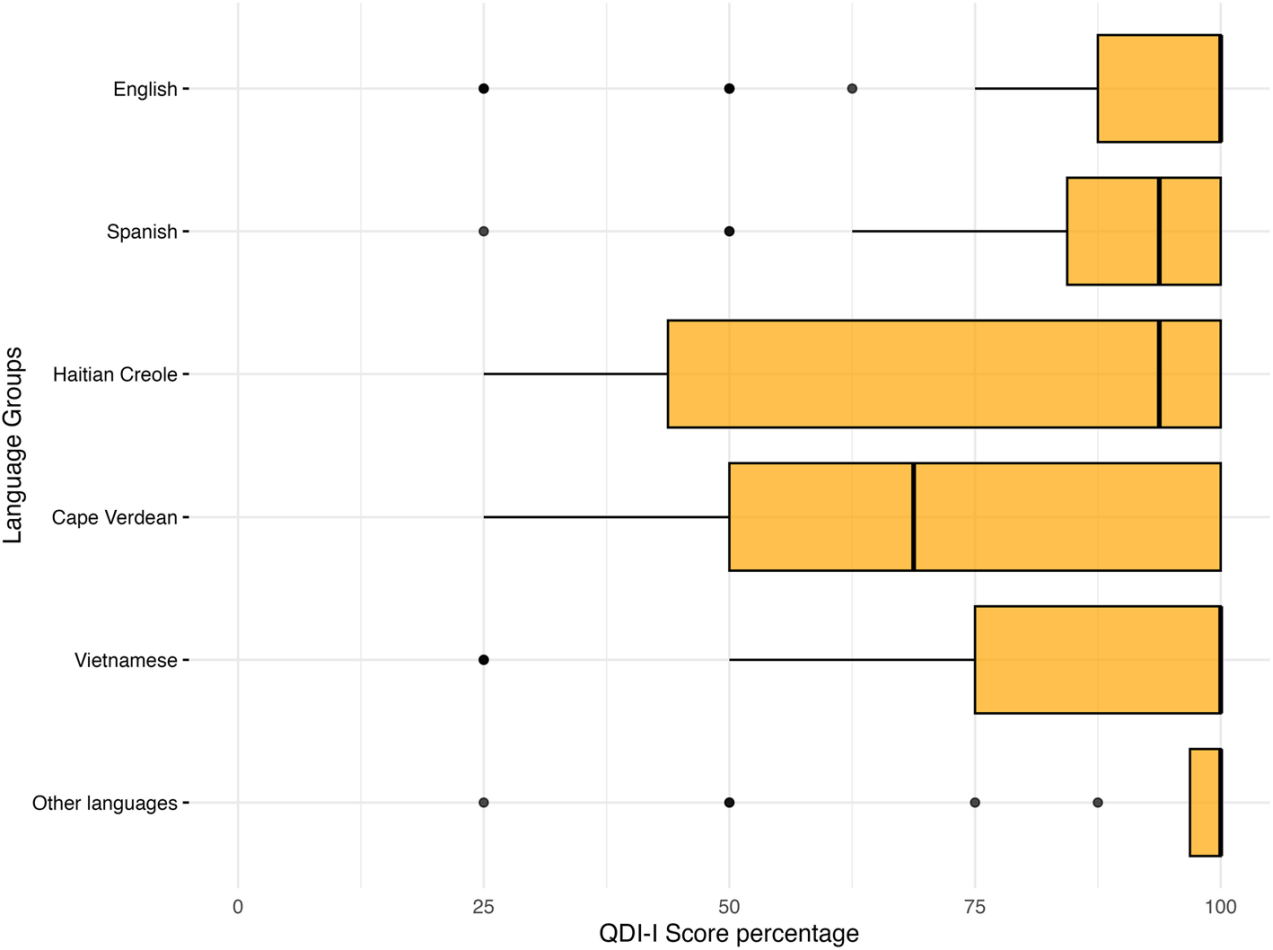
Box plot of QDI-I score for return precautions comparing English cohort with language groups within non-English language preference cohort

The overall QDI-I score did not differ significantly between the five language groups and the English cohort, but there was a trend toward higher quality instructions in the Vietnamese and Other language categories (Table 3). Comparing each QDI-I domain, both Haitian Creole and Cape Verdean Creole speakers received return precautions of inferior quality compared to the English cohort (2.95 and 2.88 vs 3.55, or 73.8% and 72.0% vs 88.8%; *p* = 0.006 and *p* = 0.001 respectively; Fig. 1).

### Comparison by intensity of self-management

Within the high-intensity self-management diagnoses, patients with NELP received shorter discharge instructions (181 words vs. 213 words, *p* = 0.048; Appendices 6 and 7). There were no significant differences in reading level or QDI-I score.

Within the low-intensity self-management diagnoses, those with NELP had similar word count and reading level as the English cohort. While their overall QDI-I scores were similar (16.68 NELP vs. 16.63 English, or 69.5% vs. 69.3%; *p* = 0.094), return precautions of those with NELP were significantly lower (3.02 vs. 3.49, or 75.5% vs. 87.3%; *p* = 0.024).

## Discussion

Written discharge instructions are an evidence-based practice to improve the transition home after medical hospitalization. While research suggests that written discharge instructions are integral to patient understanding and effective self-care, the few studies that have attempted to assess their quality have been limited and have excluded an analysis of disparities by language. In this analysis of 200 hospital discharge instructions across a variety of linguistic groups—English and five sub-groups with NELP—we found important areas for improvement in terms of linguistic accessibility, readability, and quality of content.

We found that only 8% of patients in the NELP group, most of whom were from minoritized racial and ethnic groups in our sample, received their personalized discharge instructions in their preferred language as compared to 100% in English group. For comparison, a quality improvement project at a large urban pediatric hospital which aimed to increase the provision of language-concordant discharge instructions reported a baseline of 18% [31]. While anecdotally such inequities are nearly universal at U.S. hospitals (and many other countries), prior surveys of hospitals have not collected data on the topic [32]. This systemic inequity is driven by logistical challenges to providing language-concordant discharge instructions, such as the typical delay to have documents translated and writing personalized discharge instructions before discharge, as well as the financial cost. Novel solutions are needed to address this inequity. While artificial intelligence is a promising way to produce real-time translations, studies show these methods are not yet reliable enough to be used as part of clinical care [33].

We did not find a statistically significant difference in the quality of discharge instruction content between those with English and non-English language preference except for return precautions, which were less thorough for those with NELP (*p* = 0.013). We believe it is premature to declare a lack of disparity in the quality of discharge instructions by language in other domains, especially as the clinical significance remains unexplored. First, since the QDI-I scale did not integrate into the numerical score whether instructions were language concordant, most patients with NELP could not functionally receive the written content due to language barriers. Second, our analysis was conducted in a single safety-net hospital that is nationally recognized as a leader in health equity. Providers who elect to work at this hospital are more likely to spend additional time and effort to overcome communication barriers, which may not represent average provider behavior. This theory is supported by the fact that patients with NELP received equal quality scores but slightly shorter discharge instructions, suggesting that providers used brevity as an approach to improve communication [34]. Third, aggregate analysis may mask differences in quality by language group that we could not detect due to sample size per language group [35].

The sub-group analysis by language group pointed to potential trends that should be evaluated in future studies. We found that the overall QDI-I score was lower for the Haitian Creole and Cape Verdean Creole speakers, though the differences did not reach statistical significance. As both are creole languages—meaning they originate in spoken form as a mix between other languages—this may reflect an actual or perceived difference in the likelihood of patients being able to read written instructions. In contrast, Vietnamese speakers had a higher overall QDI score than English speakers (though still not statistically significant), which may be due to more involvement of English-speaking family members in medical care [36]. A better understanding of trends specific to each language group could help tailor improvements to the hospital discharge process, such as intensive bedside education or investment in professional translation, to the needs of specific groups.

A strength of our analysis was our approach of measuring reading level. Prior studies have applied reading level calculators to discharge instructions without editing. Similar to prior research, we found that most hospital discharge instructions exceeded the reading level recommended by professional organizations [37, 38]. For example, the American Medical Association suggests that patient materials target a sixth-grade reading level. However, we found that average grade level for discharge instructions in both the English and NELP cohorts was equivalent to an eighth to ninth grade on the FKGL. By the NDCRS, only 49.0% of instructions were at an eighth-grade level in the English cohort and even fewer (38.0%) in the NELP cohort. While this difference was not statistically significant, the impact of low readability is likely greater for those who face language barriers.

We would like to highlight other limitations of our study. Because so few patients with NELP received language-concordant discharge instructions, our findings may not be applicable to the rare health system that offers translation. The diagnoses we selected for sampling discharge records were grouped into high- versus low-intensity self-management diseases as a proxy for complexity of discharge instructions but are somewhat subjective. Also, the absolute number of records reviewed per diagnosis and non-English language group was relatively small (*n* = 20 each) thus limiting statistical certainty of our findings. In addition, the QDI-I scale was based on existing evidence and guidance from professional societies but was not vetted with patients to evaluate their preferences and satisfaction with discharge instructions. Such efforts would strengthen the QDI-I, along with a study to formally validate the tool, which is currently underway.

## Conclusion

This study is the first to examine disparities in the provision of personalized written instructions at hospital discharge. Our findings show that only 8% of patients with NELP receive instructions in their preferred language; further research to understand the link between this and post-discharge disparities previously found in patients with NELP are needed. We hope these results prompt additional research on inequities in the hospital discharge process, particularly as patients with NELP have been historically excluded from evidence-based transitional care interventions.

## Supplementary Information

Supplementary Material 1: Appendix 1. Standardized template for providers to write personalized hospital discharge instructions. Appendix 2: Description of reading scores and methods for standardizing patient instructions analyzed prior to calculation of reading scores. Appendix 3: Quality of Discharge Instructions-Inpatient (QDI-I) tool. Appendix 4: Box plot of word count for English cohort and groups with non-English language preference.

## Data Availability

The spreadsheet of de-identified raw data and analysis is available on request of the corresponding author.

## Abbreviations

AVS: After visit summary
EMR: Electronic medical record
LEP: Limited English proficiency
NELP: Non-English language preference
