# Supplementary material for: Evaluating the quality and equity of patient hospital discharge instructions
Source: BMC Health Serv Res. 2025 Feb 21;25:291. doi: 10.1186/s12913-025-12410-8 (PMC11844009; doi:10.1186/s12913-025-12410-8)
Supplement: Supplementary file 1 — Supplementary Material 1: Appendix 1. Standardized template for providers to write personalized hospital discharge instructions. Appendix 2: Description of reading scores and methods for standardizing patient instructions analyzed prior to calculation of reading scores. Appendix 3: Quality of Discharge Instructions-Inpatient (QDI-I) tool. Appendix 4: Box plot of word count for English cohort and groups with non-English language preference. [file 12913_2025_12410_MOESM1_ESM.docx]

**Appendix 1: Standardized template for providers to write personalized hospital discharge instructions**

You were admitted to the hospital because ***

Instructions when you leave the hospital: ***

What to watch for: ***

Important changes to your medications: ***

Thank you for chosing Boston Medical Center.

**Appendix 2: Description of reading scores and methods for standardizing patient instructions analyzed prior to calculation of reading scores**

*Methods*

Readability calculators

The New Dale-Chall(NDC) readability formula was used as the word list (i.e. list of 3000 words that 4^th^ grader found familiar) was updated with more scientific, technical and abstract words (e.g. channel, capsule, blastoff) that were not part of the original dale chall list, which contained more rural, farm, or dated words (e.g. cackle, cluck, stump, christen). While both the new and old versions both contain 3000 words, the new version technically contains more words than 3000 as inflected words are groups together rather than separately, as in the old version. NDC formula calculates a cloze score based on the number of difficult words and average sentence length, and ranges from 58 and above for easiest passages and 10-15 and below for most difficult (Chall, JS., 1995).

Flesch Reading Ease (FRE) test and Flesch-Kincaid Grade Level (FKGL) were also used as it is commonly recommended to evaluate literacy level in health-related material (Zhou, S., 2017). FRE test uses the total words, sentences, and syllabus to provide a score between 0 to 100. Shorter sentences and syllabus would provide a higher score on the FRE test and indicate easier readability. Flesch-Kincaid Grade Level (FKGL) uses the same variables as FRE but calculates a grade level.

Text editing procedures

Before calculating the reading score, we edited patient instructions for content that we deemed important but skews these scores. This included removing numbers (for dates, addresses, vitals, and dosages), specific medication names (both generic and brand names), and medical terms when the patient friendly term was also used. Periods were placed at the end of each line of bulleted text to avoid run-on sentences. Symbols (e.g. “>” or “->”) that were placed to help with flow of the summaries were also removed. The revised summaries were pasted onto the online calculators to obtain scores for Flesch Reading Ease test^[[1]](#footnote-1)^ and New Dale-Chall readability formula^[[2]](#footnote-2)^. The decision to meticulously revise each summary was due to the sensitivity and limitations of each automated calculator (Jindal, P., 2017).

These numerical values of FRE and NDC were used to correlate to its grade levels in each of the readability calculators, FKGL and New Dale-Chall reading levels (NDCRL). While NDCRL refers to reading levels, each level correlates to the respective grade (e.g. reading level 1 = grade level 1). These levels were then categorized based on the American Medical Association (AMA) and National Institutes of Health (NIH) recommendations that patient materials should be written in sixth and eighth grade reading levels, respectively. However, FKGL categorizes 8-9^th^ grade as one group, while NDCRL categorizes 7-8^th^ as one group. Therefore, FKGL and NDCRL required separate categorical groups to accommodate these differences while keeping in line with the national recommendations for reading levels of patient materials.

- Jindal, P., & MacDermid, J. C. (2017). Assessing reading levels of health information: uses and limitations of flesch formula. *Education for health (Abingdon, England)*, *30*(1), 84–88. <https://doi.org/10.4103/1357-6283.210517>
- Chall JS.  *Readability Revisited: The New Dale-Chall Readability Formula.* Cambridge, MA: Brookline; 1995.
- S. Zhou, H. Jeong and P. A. Green, "How Consistent Are the Best-Known Readability Equations in Estimating the Readability of Design Standards?," in *IEEE Transactions on Professional Communication*, vol. 60, no. 1, pp. 97-111, March 2017, doi: 10.1109/TPC.2016.2635720.
- Weis BD. *Health literacy: a manual for clinicians*. American Medical Association Foundation and American Medical Association; 2003
- National Institute of Health. How to write easy-to-read health materials.
- Jindal, P., & MacDermid, J. C. (2017). Assessing reading levels of health information: uses and limitations of flesch formula. *Education for health (Abingdon, England)*, *30*(1), 84–88. https://doi.org/10.4103/1357-6283.210517

**Appendix 3: Quality of Discharge Instructions-Inpatient (QDI-I) tool**

| **Quality of Discharge Instructions-Inpatient (QDI-I) Scale (Version 1)**  Total Score = sum of score for domains I – IV (out of 24*)  Scaled Score = percentage out of perfect score (4 per domain, 24 total) | | | | |
| --- | --- | --- | --- | --- |
| RAW SCORE | 1 = VERY POOR | 2 = POOR | 3 = GOOD | 4 = VERY GOOD |
| ***I. Primary Diagnosis:*** *Explanation of the key diagnoses reached to explain symptoms or justify treatment decisions; describes testing done and/or diagnoses excluded* | | | | |
| If diagnosis reached | No mention of any specific diagnosis, presenting symptom, or inpatient workup/management | States only a presenting symptom without diagnosis; no mention of inpatient workup/ management | States a diagnosis with description that explains the presenting symptom but not in both medical and patient-friendly terms | Explanation of at least one diagnosis in both medical and patient-friendly terms |
| No diagnosis reached | Same | Same | States no diagnosis reached, but does not provide explanation of key testing performed or diagnoses excluded | States no diagnosis reached and provides the most likely diagnosis or explanation of diagnoses excluded and/or testing done |
| **II. Self-management instructions:** *Directs patient how to care for himself/herself after discharge, such as diet, exercise, wound care, or checking home weights* | | | | |
|  | No self-management instructions listed | At least one instruction but with little or no specific direction or rationale | At least one instruction provided with specific direction or rationale, but missing those important for diagnosis | Thorough explanation of one or more instructions including those specific for diagnosis |
| **III. Return precautions:** *Signs and symptoms that should prompt patient to seek medical attention (either from primary care provider or emergency room)* | | | | |
|  | No return precautions provided | Generic list of return precautions | At least one return precaution that is tailored to the diagnosis but misses a key return precaution for diagnosis | More than one return precaution tailored to the diagnosis (including any that are key) and provided in patient-friendly language |
| **IV. Medication changes:** *Highlights which medications were started, stopped, or changed during the hospitalization* | | | | |
| Medication changes made | No mention of medications changes or that none were made | General statement about taking medications as prescribed, but lacks any specific management instructions | Highlights some but not all important changes; management instructions may lack completeness | Highlights all important medication changes with clear and thorough management instructions, including all high-risk medications |
| No changes made | Does not state no medication changes were made |  |  | Explicitly states there were no medication changes |
| **V. Reasons for medication changes:** *Explains rationale for any medication changes during a hospitalization* | | | | |
| Medication changes made | No indication for medications changed discussed | Minimal explanation of why some (but not all) of mediation were stopped, changed, and/or started | Partial explanation of why some (but not all) of medications were stopped, changed, and/or started | Full explanation of why all medications were stopped, changed, and/or started |
| No changes made | Excluded from score | | | |
| **VI. Recommended Follow-Up:** *Provides rationale for appointments scheduled with outpatient providers, including pending results, topics to address, and contingencies* | | | | |
|  | No mention of follow up appointments or need for them | Lists generic statement about following up with primary care or mentions that the appointment was made | Provides rationale for appointments but no mention of specific procedures or tests that may be necessary | Provides rationale for appointments with details on specific procedures or test that may be necessary including key topics to discuss per diagnosis |

**CLARIFICATIONS**

1. **Primary diagnosis**

*Separate criteria if diagnosis reached during hospitalization or not*

- *If diagnosis reached provides a diagnosis that explains the patient’s presenting symptom(s), or if no presenting symptom stated explains the important diagnosis or diagnoses reached*
- *If no diagnosis reached to explain the patient’s presenting symptom, a description of what was ruled out during the hospitalization making it safe for patient to be discharged*

1. **Self-management instructions**

- *Exclusion criteria: medication instructions (start, stop, dosing, timing), generic instructions to call or see PCP/specialist, specific info on recommendations for outpatient work up (which fall under V. Recommended follow up)*
- *Inclusion criteria: instructions on wound care, habits to avoid (“avoid alcohol”), diet (“low salt” or “fluid restriction”), weight bearing, exercise or rest instructions, checking blood sugar (and management of specific values), following weights, contingencies on when to call the doctor (e.g. managing specific values of blood glucose levels)*

1. **Return precautions**

- *Generic return precautions = a standard list of symptoms covering at least 4 organ systems with at least one symptom that is clearly unrelated to the primary diagnosis or presenting symptom. (highest score this type can receive is a 2)*
- *Tailored return precautions = a list of symptoms unique to this patient’s specific diagnosis or case (considering that some symptoms may be ongoing at the time of discharge and do not require new work up)*

1. **Medication changes**

*Clear guidance on how to change them includes dose, timing, tapering, and adjustments based on labs (ex: INR) or biometrics (ex: weight gain = increase furosemide dose). “High-risk” medications and instructions include anticoagulants, antibiotics, steroids, narcotics, and hypoglycemics.*

*Special cases*

- *Discrepancy* in “start” medication between AVS and discharge med rec list, regardless of the significance of medication to primary diagnosis = 1
- If no medication changes during hospitalization
  - 1 = does not state anything about medications. A generic “continue all medications” without other statements about med
  - 4 = states explicitly there were no medication changes or states to continue medications relevant to primary diagnosis

1. **Reasons for medication changes**

**Domain not graded if no medication changes made to pre-admission regimen on discharge.*

1. **Recommended follow-up**

- *Inclusion criteria: Testing or procedures recommended at time of discharge or recommendation for specific topics to discuss or review with PCP*
- *Exclusion criteria: Symptoms that should warrant seeking emergency care = return precautions, indications only to adjust a specific medication based on conversation with PCP/specialist or contingencies on when to call the doctor = self-management, reference to follow up with clinic only about medication adjustment = medication changes*

**Appendix 4: Box plot of word count for English cohort and groups with non-English language preference**


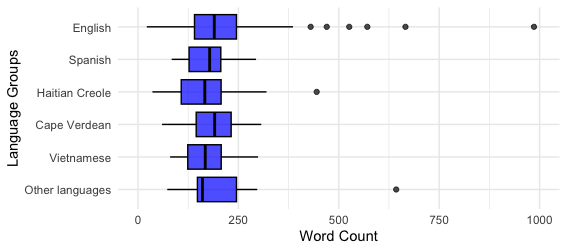


**Appendix 5: Boxplot of the Flesch Reading Ease Score (FRES) for the English cohort and the non-English language cohort by language group**


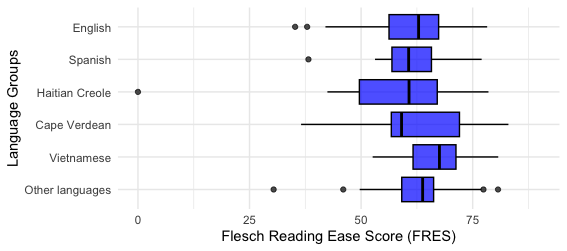


| **Appendix 6: Comparison of Content and Readability of Patient Discharge Instructions by Low and High Intensity Self-Management Groups Between NELP and English cohorts** | | | | | | |
| --- | --- | --- | --- | --- | --- | --- |
| **Outcome** | ***Low Intensity Self-Management***  ***(n=100)*** | | | ***High Intensity Self-Management***  ***(n=100)*** | | |
|  | **English** | **NELP** | **p-value** | **English** | **NELP** | **p-value** |
| Language concordant: %, n | 100%, 50 | 10%, 5 | <0.001 | 100%, 50 | 6%, 3 | <0.001 |
| Word count: median, IQR | 178 ,117.75 | 166.5, 82.75 | 0.59 | 213, 91.50 | 181, 96.25 | *0.048* |
| Flesch reading ease score: median, IQR | 60.09, 11.98 | 61.34, 12.21 | 0.63 | 63.62, 9.35 | 63.31, 14.79 | 0.71 |
| New Dale-Chall Reading Levels |  |  | 0.55 |  |  | 0.23 |
| Easier than recommended: 1- 4^th^ grade (%,n) | 0, 0% | 0, 0% |  | 0, 0% | 0, 0% |  |
| Recommended level: 5-8^th^ grade (%,n) | 24,48% | 20, 40% |  | 25, 50% | 18, 36% |  |
| Harder than recommended: 9^th^-college grads (%,n) | 26,52% | 30, 60% |  | 25, 50% | 32, 64% |  |
| Overall QDI-I score: mean, SD, %* | 16.63, 2.67  69.3% | 16.68, 3.63  69.5% | 0.94 | 17.58, 2.72  73.3% | 17.45, 2.82  72.7% | 0.82 |
| I. Primary diagnosis | 3.29, 0.68  82.3% | 3.31, 0.62  82.8% | 0.88 | 3.4, 0.60  85.0% | 3.42, 0.59  85.5% | 0.87 |
| II. Self-management | 1.93, 0.98  48.3% | 1.94, 1.05  48.5% | 0.96 | 2.26, 1.15  56.5% | 2.2, 1.10  55.0% | 0.79 |
| III. Return precautions | 3.49, 0.87  87.3% | 3.02, 1.16  75.5% | *0.024* | 3.61, 0.67  90.3% | 3.42, 0.93  85.5% | 0.25 |
| IV. Medication changes | 2.86, 1.35  71.5% | 3.09, 1.34  77.3% | 0.39 | 3.41, 1.07  85.3% | 3.31, 1.1  82.8% | 0.65 |
| V. Reason for medication changes** | 2.78, 1.04  69.5% | 2.83, 1.07  70.8% | 0.82 | 2.40, 1.10  60.0% | 2.73, 1.17  68.3% | 0.15 |
| VI. Recommended follow-up | 2.38, 0.90  59.5% | 2.57,0.88  64.3% | 0.29 | 2.46, 0.92  61.5% | 2.32, 0.84  58.0% | 0.43 |
| For categorical variables, p-values calculated using Fisher’s exact test. For continuous variables, the Mann-Whitney test was used for word count, FRES and DCRS outcomes; the two sample t-test was used for QDI-I scores  *Percentage represents score relative to a perfect score. For example, a raw total score of 18 would be 18/24 = 75%  **Reason for medication changes only applied to those changed during admission; if no medications changed this domain was not score and the total score was scaled to 24 (by multiplying by 1.2)  IQR = interquartile range; NELP = non-English language preference; QDI-I = Quality of Discharge Instruction-Inpatient scale; SD = standard deviation | | | | | | |

**Appendix 7: Box plot of word count among high (a) and low (b) intensity of self-management diagnoses in the English and non-English language preference cohorts**


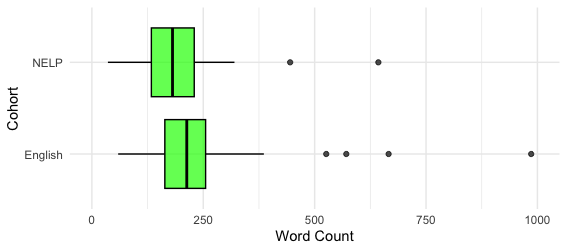


A

B


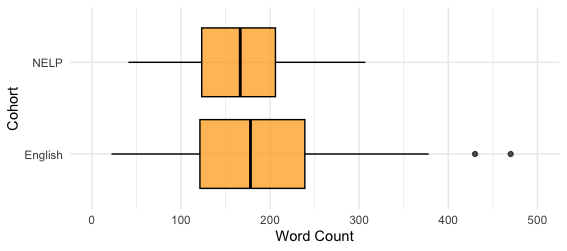


1. <https://charactercalculator.com/flesch-reading-ease/)> [↑](#footnote-ref-1)
2. <https://readabilityformulas.com/calculator-dale-chall-formula.php#formulaResults>) [↑](#footnote-ref-2)
